# Enucleation: a possible mechanism of cancer cell death

**DOI:** 10.1111/jcmm.12271

**Published:** 2014-03-14

**Authors:** Virgil Paunescu, Florina M Bojin, Oana I Gavriliuc, Elena A Taculescu, Robert Ianos, Valentin L Ordodi, Vlad F Iman, Calin A Tatu

**Affiliations:** a Department of Functional Sciences, University of Medicine and Pharmacy “Victor Babes” TimisoaraTimisoara, Romania; b Center for Transplant Immunology, Clinical Emergency County Hospital TimisoaraTimisoara, Romania; c Faculty of Industrial Chemistry and Environmental Engineering, “Politehnica” University of TimisoaraTimisoara, Romania; d Laboratory of Magnetic Fluids, Center for Fundamental and Advanced Technical Research, Romanian Academy - Timisoara BranchTimisoara, Romania

**Keywords:** cell enucleation, cancer cell death, Fe_3_O_4_ nanoparticles, tumour cells

## Abstract

There are few major morphologies of cell death that have been described so far: apoptosis (type I), cell death associated with autophagy (type II), necrosis (type III) and anchorage-dependent mechanisms—anoikis. Here, we show for the first time a possibly novel mechanism inducing tumour cell death under *in vitro* conditions—enucleation. We pursued the influence of colloidal suspensions of Fe_3_O_4_ nanoparticles on tumour cell lines (SK-BR-3 and MCF-7 breast cancer cell lines) grown according to standard cell culture protocols. Magnetite nanoparticles were prepared by combustion synthesis and double layer coated with oleic acid. Scanning and transmission electron microscopy revealed that tumour cells developed a network of intracytoplasmic stress fibres, which induce extrusion of nuclei, and enucleated cells die. Normal adult mesenchymal stem cells, used as control, did not exhibit the same behaviour. Intact nuclei were found in culture supernatant of tumour cells, and were visualized by immunofluorescence. Enucleation as a potential mechanism of tumour cell death might open new horizons in cancer biology research and development of therapeutic agents capable of exploiting this behaviour.

## Introduction

Nanoscale materials are used in diverse biomedical applications, while toxicological studies suggest that nanoparticles could induce adverse health effects. The interaction of nanoparticles with biological systems, including living cells, has become one of the important areas of collaborative research between materials science and cellular biology [1–3]. Nanoparticles are of similar size to typical cellular components and proteins, and thus may bypass natural mechanical barriers, reaching the intracellular space [4]. Once the particles are endocytosed, they may be degraded in the endolysosomal compartment, or can trigger binding of nanoparticles to intracellular targets, thus causing disturbances in cellular signalling, motility and metabolism [4].

In order to maintain homoeostasis in multicellular organisms, a balance between cellular proliferation and death should be maintained. During the tumourigenesis process, the cancer cells proliferate without control, escaping the regulatory mechanisms of cellular death. Several types of cell death have been described: apoptosis (type I), cell death associated with autophagy (type II), necrosis or oncosis (type III), mitotic catastrophe, anoikis, excitotoxicity, Wallerian degeneration and cornification of the skin [5,6]. However, it is important to note that other forms of programmed cell death have been described and other forms of cell death may yet be discovered [7–9].

This study aimed to investigate the effects of Fe_3_O_4_ nanoparticles (magnetite nanoparticles – MNPs) on tumour cells cultivated *in vitro*, using scanning (SEM) and transmission electron microscopy (TEM) and immunocytochemistry.

## Materials and methods

### Cell culture

SK-BR-3 and MCF-7 (breast cancer) cells were procured from American Type Culture Collection (ATCC, Manassas, VA, USA) and further maintained and expanded in McCoy's 5A medium (Gibco BRL, Invitrogen, Carlsbad, CA, USA) supplemented with 10% foetal calf serum (FCS; PromoCell, Heidelberg, Germany) and 1% Penicillin/Streptomycin mixture (Pen/Strep, 10,000 IU/ml; PromoCell) in standard incubator conditions, at 37°C in a humidified and 5% CO_2_ atmosphere. Cells were seeded at 7000 cells/cm^2^ in appropriate well plates and allowed to attach for 24 hrs previous to MNPs addition.

Human adult mesenchymal stem cells (MSCs), used as control, were cultured and expanded in *alpha*-minimum essential medium (MEM; Gibco BRL, Invitrogen), supplemented with 10% FCS (PromoCell) and 2% Pen/Strep (10,000 IU/ml; PromoCell). Mesenchymal stem cells were further subjected to the same experimental conditions as the tumour cell lines.

### Scanning electron microscopy

Scanning electron microscopy was performed for identification of morphological changes in tumour cell lines induced by the MNPs colloidal suspension. Cells were cultured at 7000 cells/cm^2^ in 24-well format cell culture inserts (BD Labware Europe, Le Pont De Claix, France) and colloidal suspensions were added in different concentrations. Forty-eight hours after colloidal suspensions addition, culture media was removed, cells were pre-fixed for 1 hr with 2.5% buffered glutaraldehyde (in PBS; Sigma-Aldrich Company, St. Louis, MO, USA), rinsed three times in PBS, and the 0.4 μm pore-sized membranes were detached from the culture inserts. For better image quality, cells fixed on the membranes were sputter-coated with platinum-palladium and examined with a FEI Quanta 3D FEG electron microscope (FEI Company, Eindhoven, NL, USA) generating digital electron micrographs. Each experiment was repeated five times, and the enucleation was quantified on ten individual SEM fields/experiment.

### Transmission electron microscopy

Tumour cells ultrastructural morphology was assessed by TEM, 48 hrs after addition of colloidal suspensions in different concentrations (ranging from 6.5 × 10^13^ to 6.5 × 10^15^ MNPs/ml of cell culture media). Cells were prefixed for 1 hr with glutaraldehyde (2.5% in PBS), rinsed three times in PBS, and post-fixed for 1 hr in osmium acid (2% in PBS). Dehydration was done in graded acetone in distilled water dilutions, followed by infiltration with Epon resin. Sections of about 100 nm, obtained on a diamond knife (Diatome) with Leica UC6 ultramicrotome (Leica Microsystems Inc., LKB-II, Wetzlar, Germany) were post-stained with lead citrate and uranyl acetate. The grids were examined with a FEI Tecnai 12 TEM (FEI Company).

### Cytochemistry

Extruded nuclei of the SK-BR-3 cell culture supernatant were studied by using the 4′, 6-diamino-2-phenylindole (DAPI) staining technique. SK-BR-3 culture supernatants were centrifuged for 7 min. at 500 × g and 4°C, the pellet was re-suspended in 200 μl of PBS and subsequently cytospun on microscope slides for fluorescence staining. After permeabilization and fixation with methanol for 10 min. at −20°C, the slides were stained with 1 mg/ml DAPI (Sigma-Aldrich) for 5 min. in the dark and then washed three times with PBS. Microscopy analysis was performed on a Nikon Eclipse E800 microscope (Nikon Instruments Inc., Melville, NY, USA) equipped with adequate fluorescence filters.

## Results

We investigated the influence of colloidal suspensions of magnetite (Fe_3_O_4_) nanoparticles (MNPs) on SK-BR-3 and MCF-7 breast cancer cell lines cultivated under *in vitro* conditions. The MNPs used for the preparation of colloidal suspensions were synthesized by using a new version of the combustion method [10]. After coating the nanoparticles with a double layer of oleic acid, the stabilized nanoparticles were dispersed in PBS (Sigma-Aldrich), and TEM analysis conducted on samples of 15–20 nm (data not shown).

**Figure 1:**
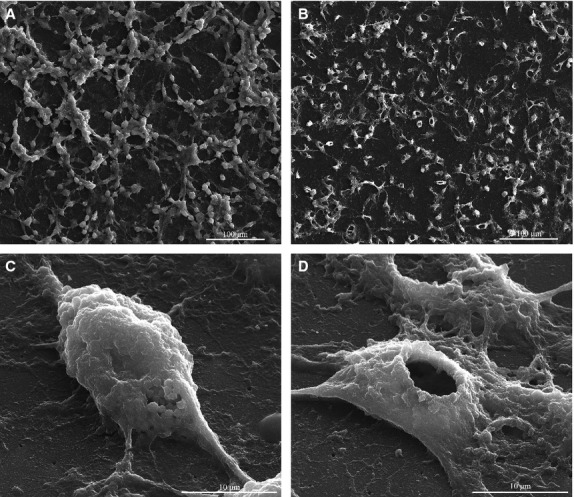
Scanning electron microscopy images of SK-BR-3 cells before and after the treatment with colloidal suspensions: (A and C) control (untreated) SK-BR-3 cells; (B and D) SK-BR-3 cells treated with MNP suspension for 48 hrs.

**Figure 2:**
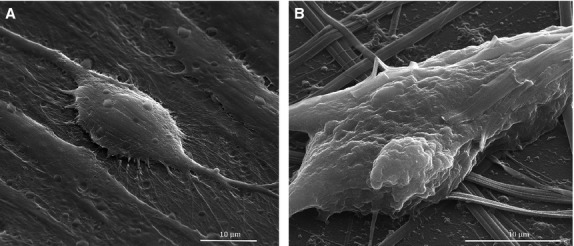
Scanning electron microscopy images of MSCs before and after the treatment with colloidal suspensions: (A) control (untreated) MSCs cells (3000×), (B) MSCs cells treated with MNPs suspension (5000×).

Morphological characteristics of untreated SK-BR-3 cells are depicted in Figure1A and C, showing the round shape, small diameter and cluster-like growth of this cell type *in vitro*, forming a network-like pattern of cellular elongations and contact points, with increased deposition of extracellular matrix within the resulted mesh. When left in contact with MNP for 48 hrs, SK-BR-3 tumour cells exhibited an unusual behaviour, extruding the nucleus, so that the cells were enucleated (Fig.1B and D). Based on quantification results obtained on 10 SEM fields/experiment, on average 90% of the tumour cells exhibited the loss of nucleus. To our knowledge, this is a novel biological phenomenon revelled for the first time. MCF-7 cells presented a very similar pattern of behaviour to SK-BR-3 cells, while the normal MSCs did not suffer the enucleation process (Fig.2A and B).

Transmission electron microscopy, performed on SK-BR-3 adherent on cell culture inserts revealed large nuclei, multiple lipid vacuoles, well-represented endoplasmic reticulum distributed along the entire cytoplasm, polyribosomes and mitochondria, demonstrating the intense metabolic processes occurring in these cells.

**Figure 3:**
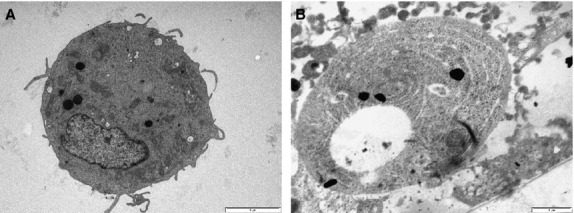
Transmission electron microscopy of SK-BR-3 cells before treatment (A) and 48 hrs after MNPs exposure (B).

When MNPs were added to the cell culture media, most of the SK-BR-3 (>90% of the cells, counted on five different TEM images) cells appeared without nuclei and fewer of the cytoplasmic structures were demonstrated, while a spiral-like concentrically oriented pattern was distinguished within the entire cytoplasm, representing stress fibres inducing the morphological changes of the cytoskeleton (Fig.3A and B). This enucleation phenomenon occurring in SK-BR-3 tumour cells treated with MNPs, while not revealed in human MSCs, might indicate a certain degree of toxic selectivity of these nanoparticles with respect to tumour cells only.

The lowest MNPs concentration to induce the enucleation process at 48 hrs was 6.5 × 10^13^ MNPs/ml of culture media, when about 90% of the tumour cells lose their nucleus. At the same concentration, the enucleation process starts insignificantly after 12 hrs, but after 24 hrs, about 30% of the cells become enucleated. Similar time-dose responses were observed by increasing MNPs concentration up to the maximum level (6.5 × 10^15^ MNPs/ml).

**Figure 4:**
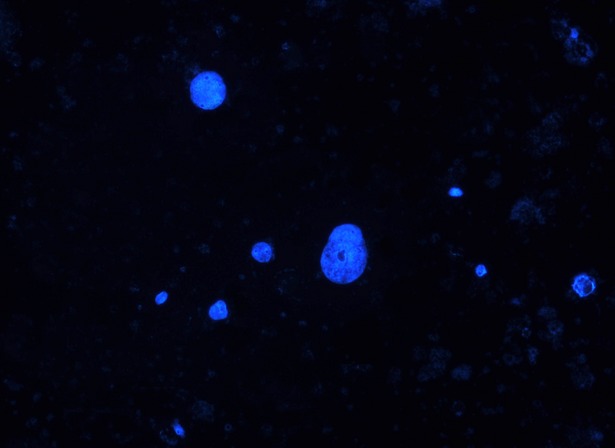
Round shape intact nuclei from culture supernatant of SK-BR-3 cells suffering the enucleation phenomenon (Magnification 200×).

We further analysed the presence of extruded nuclei within the culture supernatant. Intact nuclei were found on the slides, together with cellular residues and dead cells (Fig.4). Nuclear morphologies showed a high degree of pleomorphism. Compared to untreated cells, MNPs-treated SK-BR-3 showed on average a 10-fold increase in the counted bare nuclei per microscopy field.

## Conclusion and discussion

The enucleation phenomenon we described in the case of SK-BR-3 and MCF-7 tumour cells treated with combustion synthesized nanoparticles might reveal a novel mechanism for cancer cell death, occurring under certain environmental conditions. As a physiological process, enucleation has been thoroughly documented in the erythroid terminal differentiation process [11]. However, the tumour cell lines enucleation process described by us is most likely a non-physiological process and mechanistically unrelated to erythroblast enucleation. On the other hand, in the presence of combustion synthesized nanoparticles normal MSCs developed anchorage structures, which made them more resistant to the chemical stress, and more important, did not suffer the enucleation phenomenon.

However, additional research will be needed to document the specificity and universality of the enucleation phenomenon in the case of tumour cells and to what extent such a mechanism could develop under *in vivo* conditions.
